# Beyond conventional immune checkpoints: the LILRB axis as a dual regulator of tumor and immune cell states

**DOI:** 10.1186/s13046-026-03796-2

**Published:** 2026-08-01

**Authors:** Alessandro Scagliotti, Ivan Depetro, Giorgia Tiberi, Paula Ariadna Diez-Villegas, Ermes Candiello, Paola Cappello

**Affiliations:** 1 Department of Molecular Biotechnology and Health Sciences, University of Turin, Turin, Italy; 2 Molecular Biotechnology Center “Guido Tarone”, University of Turin, Turin, Italy

**Keywords:** Immune checkpoints, LILRB family, Immunosuppression, Cancer fitness

## Abstract

Immune checkpoint blockade targeting cytotoxic T lymphocyte–associated protein 4 (CTLA-4) and programmed cell death protein 1 (PD-1)/PD-L1 has transformed cancer therapy. However, limited response rates and frequent resistance highlight additional mechanisms of immune suppression within the tumor microenvironment (TME), particularly those driven by myeloid and innate immune compartments. The leukocyte immunoglobulin-like receptor B (LILRB) family has emerged as a central regulatory axis integrating immune and tumor cell–intrinsic programs. Beyond restraining immune activation, LILRB receptors reprogram myeloid cells toward tolerogenic states and, when expressed by tumor cells, directly sustain proliferation, metabolic adaptation, angiogenesis, and immune evasion. This dual functionality positions the LILRB axis as a system-level checkpoint linking immune suppression with tumor cell fitness. Here, we synthesize current knowledge on LILRB biology and discuss its implications for cancer immunotherapy, highlighting how targeting this pathway may overcome resistance to PD-1/PD-L1 blockade and enable more effective combination strategies aimed at reprogramming the tumor–immune ecosystem.

## Introduction

### Reframing immune checkpoints: the LILRB axis as a dual regulator of tumor and immune cell states

The clinical success of immune checkpoint blockade (ICB) has established inhibitory receptor pathways as central regulators of antitumor immunity. Targeting cytotoxic T lymphocyte–associated protein 4 (CTLA-4) and programmed cell death protein 1 (PD-1)/programmed death ligand 1 (PD-L1) has shown that releasing inhibitory constraints on T cells can induce durable tumor regression in a subset of patients. However, most patients fail to achieve long-term benefit, and both primary and acquired resistance remain major clinical challenges [1]. These limitations indicate that the current checkpoint paradigm, which is largely centered on T cell-intrinsic inhibition, captures only part of the regulatory complexity of tumor-associated immune suppression.

Recent advances have highlighted the dominant role of the tumor microenvironment (TME) in shaping immune responses, with myeloid cells emerging as key orchestrators of immune dysfunction. Tumor-associated macrophages, dendritic cells, and myeloid-derived suppressor cells actively suppress T cell activity, impair antigen presentation, and promote tissue remodeling and tumor progression [2]. These observations have led to the recognition that immune regulation in cancer extends beyond classical lymphoid checkpoints and involves additional inhibitory pathways embedded within innate immune circuits. Among these, the leukocyte immunoglobulin-like receptor (LILR) family, and particularly the inhibitory LILRB subfamily, has emerged as a critical regulatory axis. The LILR family comprises class I transmembrane receptors subdivided into activating (LILRA1–6) and inhibitory (LILRB1–5) members, which are distinguished by association with immunoreceptor tyrosine-based activation motifs (ITAMs) or the presence of immunoreceptor tyrosine-based inhibitory motifs (ITIMs), respectively. In mice, paired immunoglobulin-like receptor (PIR)B is the functional orthologue of human LILRB2 and LILRB3, sharing conserved structural and signaling properties, whereas gp49B1 exhibits structural and functional similarity to human LILRB4 [3].

LILRB receptors are broadly expressed across hematopoietic lineages, with prominent expression in myeloid cells, including macrophages, dendritic cells (DCs), granulocytes, and myeloid-derived suppressor cells (MDSCs), but they are also detectable in lymphoid populations such as T, B, and Natural Killer (NK) cells [3]. Upon ligand engagement, LILRB receptors signal through ITIM-dependent recruitment of phosphatases such as SHP-1 and SHP-2, thereby dampening activating pathways downstream of Fc receptors, Toll-like receptors (TLR), and cytokine receptors [4]. A defining feature of LILRB receptors is their ability to engage a broad and heterogeneous repertoire of ligands, including classical and non-classical HLA class I molecules as well as non-HLA ligands such as angiopoietin-like proteins, β-amyloid, cystatin C, serum amyloids, and semaphorins [3]. This broad ligand recognition enables LILRB receptors to integrate immune- and tissue-derived signals, positioning them as context-dependent regulators of cellular activation in inflamed and tumor-associated environments. As discussed below, LILRB receptors operate at two interconnected levels within cancer biology. At the immune-extrinsic level, they function as central regulators of myeloid cell programming, promoting tolerogenic differentiation, suppressing antigen presentation, and directly or indirectly limiting T and NK cell effector responses. At the tumor-intrinsic level, their aberrant expression in malignant cells sustains proliferative signaling, metabolic adaptation, angiogenesis, and immune evasion. This dual functionality distinguishes the LILRB axis from classical immune checkpoints and suggests that it acts as a coordinated system linking immune suppression with tumor cell fitness. PD-1 has also been reported to be expressed by tumor cells; however, its intrinsic role in that setting remains poorly defined [5]. Rather than functioning as isolated inhibitory receptors, LILRBs appear to organize a broader regulatory network that integrates signals across immune and non-immune compartments. In this framework, they can be viewed as immune checkpoints with system-level activity, capable of simultaneously modulating the tumor microenvironment and tumor cell behavior.

In this Review, we discuss the molecular and cellular mechanisms through which LILRB receptors regulate tumor immunity, emphasizing their dual role in immune and tumor compartments. We further explore how this axis intersects with established checkpoint pathways and how its targeting may overcome resistance to current immunotherapies. By integrating emerging evidence across different tumor types and biological contexts, we propose that the LILRB axis represents a new layer of immune regulation with important implications for cancer therapy.

### The LILRB signaling system

**Fig. 1:**
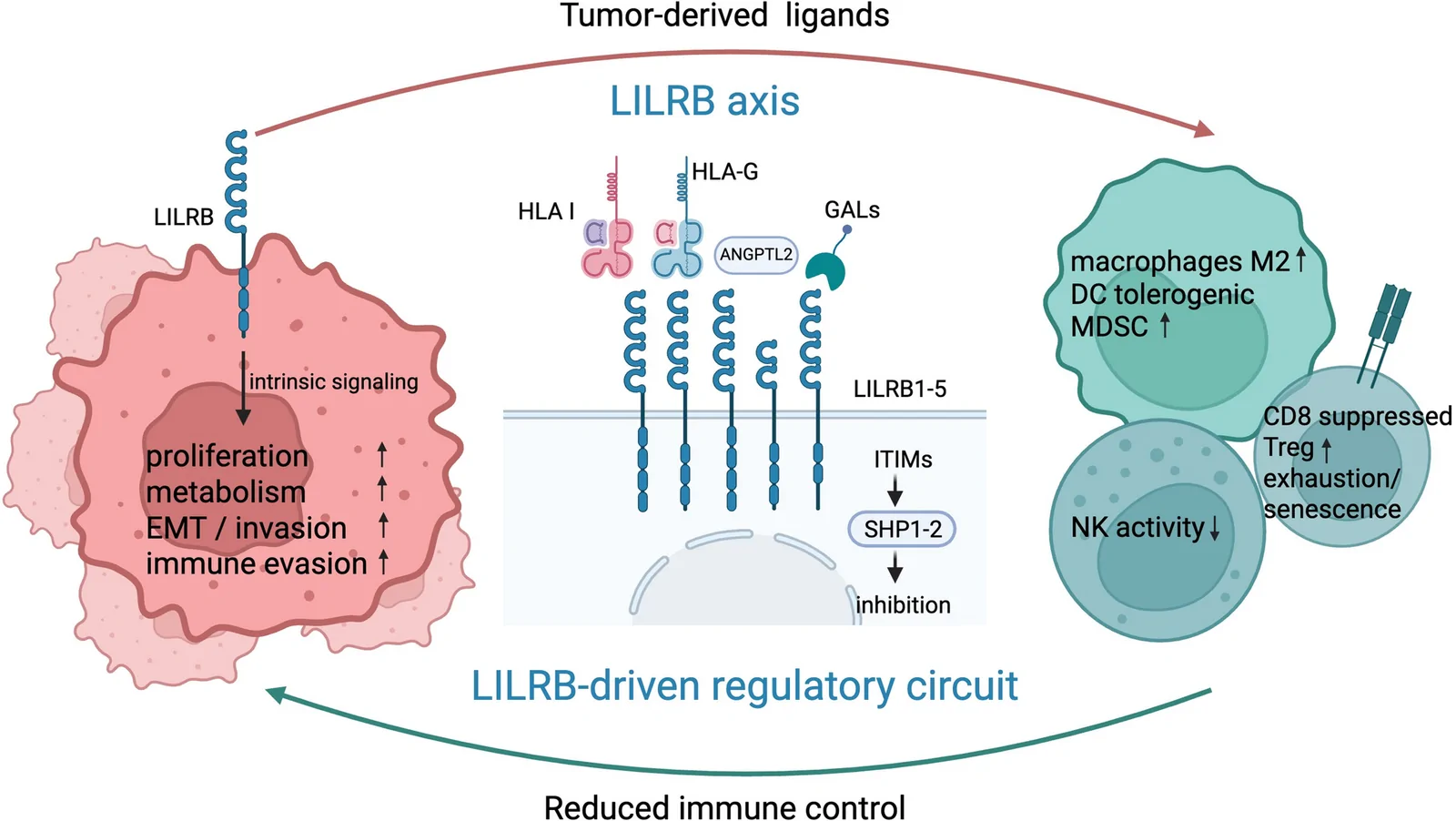
The LILRB axis is a dual checkpoint system coordinating tumor progression and immune suppression. LILRB receptors function as bidirectional regulators linking tumor cells and the tumor microenvironment. In tumor cells, LILRB signaling promotes proliferation, metabolic adaptation, invasion, and immune evasion. In parallel, LILRB engagement in myeloid cells induces tolerogenic programming, suppresses antigen presentation, and indirectly and directly inhibits T- and NK-cell effector functions. Through the integration of diverse ligands derived from tumor, stromal, and immune compartments, the LILRB axis establishes a feed-forward loop that sustains both tumor-cell fitness and immune suppression, defining a system-level immune checkpoint beyond classical T cell-centric pathways. Figure created in https://BioRender.com

LILRB receptors belong to the leukocyte immunoglobulin-like receptor family and are characterized by the presence of cytoplasmic immunoreceptor tyrosine-based inhibitory motifs (ITIMs), which mediate inhibitory signaling upon ligand engagement (Fig. 1). Following receptor clustering, Src-family kinases phosphorylate ITIMs, enabling recruitment of Src homology region 2 domain-containing phosphatase-1 (SHP-1) and SHP-2 and, to a lesser extent, Src homology 2 domain-containing inositol 5-phosphatase (SHIP), resulting in inhibition of activating pathways, including those downstream of Fc receptors, TLR, and cytokine receptors [3]. Although a conserved ITIM-dependent inhibitory property is shared across all LILRB members, their signaling outputs are not interchangeable and are highly context-dependent. As previously reviewed by *Zhang* group, non-mutually exclusive mechanisms may account for these differences. Despite the presence of conserved ITIM motifs, differences in the amino acid sequence and surrounding residues make single ITIMs functionally distinct, thereby influencing phosphorylation efficiency, phosphatase recruitment and downstream signaling [6]. Indeed, the relative contribution of SHP-1 and SHP-2 varies among LILRB family members, while LILRB4 has also been reported to recruit SHIP1, further expanding the diversity of inhibitory signaling modules [7]. As mentioned, the signaling outcome is profoundly influenced by the cellular-specific transcriptional and signaling landscape, including the relative abundance of phosphatases, kinases, scaffold and downstream proteins, thereby determining how LILRB engagement is translated into biological effects. Finally, the unique ligand repertoire of each LILRB, together with ligand binding to different receptor epitopes, may induce different receptor conformations and consequently different outputs. Ligand specificity differs among family members: LILRB1 preferentially recognizes β2-microglobulin-associated classical and non-classical HLA class I molecules, particularly HLA-G [3, 8], whereas LILRB2 displays a broader ligand repertoire that includes HLA molecules as well as angiopoietin-like proteins (ANGPTLs) [9], β-amyloid [10], myelin inhibitors of axonal regeneration [11], oligomerized cystatin C (a cysteine cathepsin inhibitor), serum amyloid A4 (SAA4) [12], semaphorin-4 A (SEMA-4 A) [13], and as recently discovered also claudins [14]. Although canonical LILRB signaling relies on SHP-mediated phosphatase activity, accumulating evidence indicates that ligand- and cell-type signaling can also engage non-canonical pathways, as reported for the ANGPTL2–LILRB2/PIRB axis able to induce the expression of inflammatory mediators and the phosphorylation of signaling pathways such as MAP kinase, NF-κB, and AKT, thereby modulating cellular functions beyond simple inhibition [15]. Rather than functioning through highly specific ligand-receptor pairs, LILRB receptors display ligand promiscuity, allowing them to integrate signals derived from immune cells, stromal components, and tumor cells. This is the case of LILRB4, unable to bind HLA [16], which can activate ITIM-dependent inhibitory pathways after engagement with apolipoprotein E (APOE), galectin-8, and fibronectin, often highly expressed inside the tumor microenvironment [6, 17, 18]. Different LILRB isoforms contribute to variability in ligand affinity and binding capacity. An illustrative example is LILRB3, for which early studies reported an inability to bind HLA class I molecules, whereas more recent reports have challenged this notion [19]. LILRB3 was long considered an orphan receptor; however, a specific LILRB3 isoform has recently been shown to bind the complement component C4d, in addition to HLA class I molecules, a feature otherwise shared only with LILRB2 [20]. LILRB3 has also been reported to be activated by galectin-4 and galectin-7, which are widely expressed by tumor cells and exert context-dependent pro- and immunosuppressive functions, but overall support tumor progression, adhesion, invasion, and metastasis [21, 22]. LILRB3 can also bind APOE4 [23] and a cytokeratin 8-derived ligand expressed by necrotic glandular epithelial cells [24]. Finally, besides its ability to bind HLA molecules and weakly claudins, physiological ligands of LILRB5 remain incompletely characterized [6, 14].

This signaling architecture suggests that LILRB receptors function as context-dependent regulators rather than simple inhibitory switches. The signaling outcome is further influenced by the modality of ligand engagement. In particular, ligand engagement can occur in *cis* or in *trans*. The *cis* interactions happen on the same cell surface, contributing to tonic inhibitory signaling and immune homeostasis, as thoroughly described below (Sect.  3). The *trans* interactions, instead, physiologically put in contact distinct cells to induce immune tolerance and can be co-opted by tumor cells at their favor through the expression of different LILRB ligands such as HLA-G [25]. A paradigmatic example of this *cis-to-trans* dynamic is provided by the interface between DCs and cytotoxic T lymphocytes. The constitutive *cis* interaction with HLA molecules not only promotes a tolerogenic DCs phenotype but also prevents HLA recognition by CD8 molecules. However, once the immunological synapse forms, HLA molecules are preferentially engaged in *trans* on the T cell surface, relocating the complex to the periphery of the supramolecular activation cluster, relieving the competitive block on CD8 and allowing productive TCR/CD8-HLA engagement [26]. However, the signaling cues that induce a preferential *cis* or *trans* interactions have yet to be identified, although *cis* engagement appears to represent the dominant regulatory mode. Indeed, *trans* HLA availability has been proven to fail to compensate for the loss of *cis* signaling in some cellular context [27].

Importantly, species differences between murine Paired Immunoglobulin-like receptor B (PIRB) and human LILRB receptors complicate direct translation, as multiple human receptors may be functionally compressed into a small number of murine orthologues.

### Translational considerations: human vs. murine LILRB signaling

Murine models have been instrumental in elucidating fundamental aspects of inhibitory receptor biology, and several key features of LILRB signaling are conserved between species. In particular, PIRB shares structural and functional similarities with human LILRB receptors, including ITIM-dependent recruitment of SHP-1 and SHP-2 and broadly comparable inhibitory signaling outputs. In addition, several ligand classes, including MHC class I molecules and angiopoietin-like proteins, are conserved, supporting the relevance of murine systems for mechanistic studies [3]. Nevertheless, LILRBs are primate-specific receptors encoded within the leukocyte receptor complex (LRC) on chromosome 19q13.4, whereas murine inhibitory receptors have evolved independently and therefore represent functional relatives rather than true orthologs [28].

Important differences exist at the level of receptor complexity and specificity. In humans, five inhibitory receptors (LILRB1–LILRB5) display partially overlapping but non-redundant expression patterns across immune and non-immune populations, whereas mice lack a corresponding orthologous expansion. Instead, PIRB functionally approximates primarily LILRB3 and, to a lesser extent, LILRB2, resulting in a many-to-one relationship between murine and human systems. This ambiguity is further explained by domain-level discordance: while PIRB shows the highest overall sequence identity with LILRB3, its D1D2 extracellular domains, which mediate ligand engagement, align more closely with the D1D2 domains of LILRB2. This structural correspondence has direct functional consequences, as β-amyloid oligomers bind PIRB and LILRB2, but not LILRB1 and LILRB3 [10]. Similarly, gp49B1 has been identified as the closest murine relative of human LILRB4, and was subsequently renamed accordingly [29], suggesting that the murine inhibitory receptor repertoire is more complex than initially appreciated. However, gp49B1 and LILRB4 also diverge structurally, with gp49B1 retaining only two cytoplasmic ITIMs compared to three in human LILRB4 [29]. This difference likely underlies their partially divergent ligand specificity: APOE, the principal activating ligand of LILRB4 in leukemia, engages murine PIRB but not gp49B1, while integrin avb3, originally described as a gp49B1 ligand, fails to activate LILRB4 [30].

These evolutionary differences have important implications for translational research, limiting the ability to resolve receptor-specific functions, particularly in contexts in which distinct LILRB isoforms have non-redundant roles. Because PIRB integrates functions that are distributed among multiple human receptors, findings observed in PIRB-deficient models may reflect the combined loss of signaling pathways that are mediated by distinct receptors in humans. Moreover, the broader cellular expression of PIRB may generate composite phenotypes that do not directly mirror receptor-specific perturbations in human tissues. Consequently, while murine models remain indispensable for investigating the biology of the LILRB/PIRB axis, these differences underscore the importance of integrating murine studies with human primary cells, patient-derived samples, or humanized mouse models when evaluating the biological and therapeutic relevance of the LILRB axis.

## Rewiring the tumor microenvironment

### Myeloid axis as central regulatory hub

The signaling properties are particularly relevant in the tumor microenvironment, where LILRB receptors are predominantly expressed by myeloid populations, including monocytes, macrophages, DCs, and MDSCs, where they act as central regulators of immune suppression and tolerance (Fig. 1). In monocytes, LILRBs establish a conserved inhibitory axis that restrains Fc receptor-dependent activation, suppresses antigen presentation, and enforces M2-like polarization through inhibition of ERK, NF-κB, and STAT1 signaling [27, 31]. Similar programs operate in DCs, in which sustained LILRB expression is a hallmark of tolerogenic differentiation and reduced antigen presentation. Immunoregulatory cues, including tolerogenic stimuli induced by dexamethasone [32] and rapamycin [33] and exposure to CD8^+^CD28^−^ suppressive T cells [34], maintain high LILRB levels in DCs and impair T cell priming. Engagement of HLA-G, particularly through LILRB2, activates STAT3 signaling and stabilizes DCs in an immature-like state [8]. These tolerogenic programs are physiologically reinforced at the feto-maternal interface through HLA-G-dependent LILRB engagement, which promotes the expansion of Interleukin (IL)10-producing tolerogenic DCs and type 1 regulatory (Tr1) cells [35]. During infection, however, sensing of PAMPs and DAMPs can transiently downregulate LILRB/PIRB through TLR signaling, thereby enabling APC activation and type I interferon responses [36], although bacterial and viral pathogens can also hijack this axis [37, 38]. Tumors similarly co-opt LILRB signaling to enforce an immunosuppressive myeloid program associated with poor clinical outcomes [39, 40]. Tumor-derived ligands, including secretogranin 2 and cystatin C, engage myeloid LILRBs to activate STAT3 [41] and TGFβ [12] pathways, induce LILRB/PIRB upregulation, promote M2-like polarization, and sustain tolerogenic circuits driven by MDSCs that induce Th2 and Treg responses within the TME and drive matrix remodeling and metastatic progression [42, 43]. In this context, LILRB1 and LILRB2/PIRB *cis* interactions with HLA molecules on DCs restrain cytotoxic T cell priming [26], whereas stromal components further sustain this program, as fibronectin engagement of LILRB4 promotes suppressive myeloid polarization [18]. Genetic or pharmacological disruption of the LILRB/PIRB axis reprograms myeloid cells toward inflammatory phenotypes, enhancing T cell priming and cytotoxic activity, including direct antitumor activity by MDSCs [44, 45]. Collectively, these findings identify the LILRB/PIRB axis as a central, targetable checkpoint of myeloid-driven immune suppression in cancer.

### The innate effectors: granulocytes and NK cells

While myeloid antigen-presenting cells represent the dominant axis of LILRB-mediated regulation, additional innate populations such as granulocytes, including neutrophils, eosinophils, and mast cells, also contribute to this network. Compared with myeloid APCs, however, the contribution of LILRB signaling in these populations has been less extensively investigated, and current evidence suggests a modulatory role [46–48].

In neutrophils, LILRB2 and LILRB3 suppress phagocytosis, oxidative burst, and integrin-mediated signaling [49]. Agonistic engagement of LILRB3 inhibits FcγR- and FcαR-mediated neutrophil activation [50], and similar results have been observed in PIRB-expressing neutrophils. Indeed, genetic deletion of PIRB enhances oxidative burst, granule release, adhesion to integrin ligands, and chemotaxis [51]. These observations support the functional similarities between human LILRB2/3 and murine PIRB, which are also evident in other cell types upon engagement of the common ligand HLA-G. Functionally, LILRB3 limits premature neutrophil activation, whereas LILRB2 restrains post-activation responses, and both converge on complement-dependent suppression through engagement of C3b, C4b, and C4d complement factors [20]. Pathogens have evolved mechanisms to exploit this inhibitory axis, as the β protein of *Streptococcus agalactiae* selectively binds to LILRB3, but not LILRB2, suppressing FcγR-mediated neutrophils ROS production [52]. Similarly, within the TME, tumor- and stroma- derived factors can engage LILRB receptors to promote a pro-tumoral skewing toward an N2-like neutrophil phenotype [12].

Similar effects are mediated by LILRB receptors in eosinophils, basophils, and mast cells. Human eosinophils and basophils mainly express the LILRB3 isoform [46], together with LILRB4 in basophils [48], whereas murine eosinophils express high levels of PIRB and gp49B1 [53, 54]. Human data on mast cells were obtained largely using cord blood-derived mast cells differentiated in vitro and may not fully reflect in vivo tissue-resident mast cells [55]. In mice, inhibitory signaling through PIRB and gp49B1 is more clearly established. For example, PIRB knockdown enhances eosinophil infiltration and hyperactivation in a model of allergic inflammation [56]. Notably, PIRB can also mediate activating signals in eosinophils upon leukotriene B4 stimulation through a noncanonical ITSM within its ITIM domain, thereby enabling ERK1/2 signaling [57]. Upon co-ligation with FcεRI, phosphorylated PIRB suppresses IgE-mediated activation, whereas *cis* interaction of LILRB2/PIRB with MHC class I further restrains premature mast cell responses [58]. In cancer, LILRB signaling in these innate effector populations remains incompletely defined and appears to vary across contexts. Available evidence nevertheless suggests that it can contribute to polarization toward tumor-promoting phenotypes. For example, LILRB2 engagement synergizes with TGFβ signaling and promotes pro-tumoral neutrophil differentiation, whereas LILRB1 expression has been linked to an eosinophil-associated signature and poor prognosis in glioma [12, 59].

Natural killer (NK) cells are crucial effectors of innate immunity and antitumor immune responses. Their function is tightly regulated by the balance between inhibitory and activating receptors. LILRB receptors belong to the inhibitory arm, with LILRB1 being the predominant isoform, whereas LILRB2 and LILRB3 are detected only at low levels in cancer-related contexts [60] (Fig. 1). LILRB expression is typically upregulated on NK cells in the presence of tumor [61, 62], and antagonistic anti-LILRB1 monoclonal antibodies enhance NK-cell activation and tumoricidal activity against both hematological and solid malignancies [63]. Interestingly, mature murine NK cells lack PIRB expression [64]. Natural killer T (NKT) cells, a hybrid lymphocyte population expressing both TCRs and NK receptors and typically activated by lipid antigens presented by CD1 molecules, also appear to be indirectly regulated by LILRB2 expressed on APCs. Specifically, LILRB2 interaction with CD1d markedly influences its recycling and reduces lipid-antigen presentation, thereby diminishing NKT-cell stimulation [65].

### Adaptive immunity reshaping

These innate immune programs have profound downstream consequences on adaptive immunity. As discussed above, myeloid-cell reprogramming driven by LILRB signaling suppresses CD8^+^ T cell activation and promotes Treg expansion (Fig. 1). Among the LILRB family, LILRB1 appears to be the only receptor consistently expressed on both CD8^+^ and CD4^+^ T cells, and it emerges as a hallmark of terminally differentiated antigen-specific effector/memory cells displaying high cytotoxic potential and features of cellular immunosenescence, particularly within the TME across different tumors [66, 67]. However, LILRB1^+^ CD8^+^ and CD4^+^ T cells remain sensitive to inhibition upon engagement with HLA-G molecules: both membrane-bound and soluble HLA-G markedly suppress degranulation, cytokine secretion, and proliferation of LILRB1^+^ T cells, effects that can be reversed by LILRB1 blockade [68, 69]. HLA-G is likely the principal mediator of LILRB1^+^ T-cell inhibition in cancer. Interestingly, LILRB1 and PD-1 show mutually exclusive expression patterns, with LILRB1 being expressed earlier than PD-1 during T cell stimulation [70] but reappearing at high levels in patients who fail PD-1 blockade [71]. Functional studies demonstrate that dual inhibition of PD-1 and LILRB1 synergistically restores CD4^+^ granzyme B (GzmB)^+^ T-cell proliferation, Th1 marker expression, and cytotoxicity in an MHC-II-dependent manner, highlighting LILRB1 as a non-redundant immune checkpoint [69].

LILRBs have also been implicated in the regulation of T-helper-cell differentiation. LILRB2 has emerged as a key regulator of CD4^+^ T-cell polarization. In humans, LILRB2 is selectively induced on activated CD4^+^ T cells, where it functions as a receptor for semaphorin-4 A (SEMA4A), a molecule highly expressed by DCs and B cells. Engagement of the SEMA4A–LILRB2 axis selectively drives Th2 polarization by enhancing the production of IL4, IL5, and IL13 while suppressing Th1-associated gene expression. Importantly, IL4 itself further upregulates LILRB2 on activated CD4^+^ T cells, thereby stabilizing Th2 polarization [13]. LILRB3 and its murine orthologue PIRB also play a critical role in regulating Th17 responses. Genetic ablation of PIRB in CD4^+^ T cells selectively impairs Th17 differentiation without affecting Th1 polarization by altering mammalian target of rapamycin complex 1 (mTORC1) signaling. Both insufficient and excessive mTORC1 activation disrupts Th17 fate commitment and promotes apoptosis, positioning PIRB as a critical regulator of this balance. Elevated LILRB3 expression segregates with severe ileal Crohn’s disease and ulceration, supporting a link between LILRB3 and pathogenic Th17-driven inflammation [72]. Gp49B1 is prominently expressed by immunoregulatory CD4^+^ T-cell subsets, including both FOXP3^+^ Tregs and Tr1 cells. In cancer, neoantigen vaccination induces expansion of a highly suppressive population of cytotoxic gp49B1^+^ Tr1 cells characterized by elevated GzmB expression, IL10 production, and potent inhibition of antitumor immunity. Mechanistically, gp49B1 cooperates with co-expressed SEMA4A to enforce the Tr1 suppressive program and promote immune regulation. Functional blockade of gp49B1 restores CD8^+^ T-cell function and overcomes resistance to PD1 blockade [73, 74].

Regarding the humoral branch of adaptive immunity, both mature naïve and memory B cells predominantly express LILRB1 under homeostatic conditions, whereas other isoforms are only modestly expressed. However, when infiltrating tumors such as hepatocellular carcinoma, B cells can upregulate multiple family members, including LILRB2, LILRB3, LILRB4, and LILRB5 [60]. Functionally, inhibitory LILRBs act as key checkpoints that limit B-cell activation and humoral output. Engagement of LILRB1 by HLA-G suppresses B-cell proliferation, cytokine production, class-switch recombination, and antibody secretion while skewing responses toward IL10-dominated programs and impaired memory differentiation [75, 76]. Unlike T cells, murine B cells also express PIRB and gp49B1, providing a useful model for mechanistic insight into these regulatory axes, which counteract antibody production [77, 78]. Despite this well-defined immunoregulatory framework, the role of LILRB-expressing B cells in tumor immunity remains largely unexplored, and current evidence is mainly limited to correlations between high LILRB expression and reduced B-cell infiltration in gastric cancer [79].

Collectively, these observations position LILRB receptors as upstream regulators of adaptive immune dysfunction rather than conventional T-cell checkpoints. Unlike PD-1 or CTLA-4, which are primarily induced after T-cell activation and function by restraining ongoing effector responses, LILRB receptors are constitutively expressed by myeloid antigen-presenting cells, where they shape the earliest phases of adaptive immunity. By controlling antigen presentation, co-stimulation, and myeloid-cell differentiation toward tolerogenic phenotypes, LILRB signaling determines the quality of subsequent T-cell priming and polarization before classical checkpoint pathways become engaged. In addition, direct LILRB expression on lymphocytes further modulates T-cell differentiation and effector functions in a context-dependent manner. Finally, LILRB expression is dynamically regulated by a broad range of environmental cues, including immunosuppressive mediators such as IL10, HLA-G [80], and vitamin D3 [55], as well as inflammatory stimuli including type I interferons [6], further supporting its role as a central upstream regulatory node coordinating innate and adaptive immunity.

## Tumor-intrinsic functions: LILRB receptors as regulators of cancer cell fitness

Beyond their established role as inhibitory receptors in immune cells, LILRB family members are increasingly recognized as tumor-intrinsic regulators of malignant fitness (Fig. 1). Across multiple solid tumors, LILRB receptors are aberrantly expressed by cancer cells and, as discussed below, support core hallmarks of tumor progression, including sustained proliferation, metabolic adaptation, angiogenesis, invasion, and immune evasion. Rather than acting solely as markers of aggressive disease, these receptors function as signaling hubs that integrate extracellular cues and translate them into programs that promote tumor survival and progression.

### LILRB receptors sustain proliferative and survival programs

A consistent finding across tumor models is that increased expression of LILRB receptors correlates with enhanced proliferative capacity and poor clinical outcome. Elevated tumor-cell expression of LILRB1, LILRB2, LILRB3, and LILRB4 has been reported in several types of cancer, including glioma [59, 81], lung [82], colorectal [83, 84], breast [85], gastric [86], endometrial [87], and pancreatic [88, 89] cancer. Despite isoform-specific distribution patterns, LILRBs functionally converge to sustain tumor-cell proliferation, clonogenic growth, and in vivo expansion, as shown by both loss- and gain-of-function studies. Mechanistically, LILRB-driven tumor growth converges on recurrent signaling modules, most prominently ERK1/2 [85, 90] and PI3K-AKT-mTOR [85, 91], with additional contributions from NF-kB [92] and SHP2-CAMK1-CREB [93] in selected contexts. Consistent with these functions, high LILRB expression correlates with aggressive clinicopathological features, including increased tumor size, lymph-node involvement, advanced stage, metastatic dissemination, and reduced survival [84, 90].

### LILRB signaling links tumor growth to immune escape

These mechanisms are further coupled to immune evasion and limited effective antitumor immunity. In contrast to conventional checkpoint pathways, which primarily restrain immune effector cells, LILRB receptors expressed by tumor cells directly reduce cytotoxic T-cell infiltration [83, 84], promote Treg accumulation [83], and favor the establishment of a suppressive myeloid niche [83, 93]. In breast, lung, melanoma, and colorectal models, tumor-derived LILRB2/PIRB promotes the induction of senescent T-cell phenotypes through HLA-G engagement [90]. Additional immune-evasion mechanisms include reduced antigen presentation and co-stimulation, as LILRB2 has been shown to promote degradation of HLA class I molecules [92] and induce the expression of co-inhibitory ligands such as B7-H3 [94], thereby weakening tumor-cell susceptibility to CD8^+^ T cell-mediated killing. Importantly, targeting this axis restores T-cell function and constrains tumor growth, highlighting its therapeutic potential [83]. A distinct checkpoint axis involves tumor-expressed claudins, particularly the 18.2 variant restricted to gastric, pancreatic, and biliary tract tumors, which engages LILRB2 in a bidirectional signaling loop. On one side, LILRB2 activation enhances MDSC-mediated T cell suppression and orchestrates an immunosuppressive TME at the expense of effector T and NK cells. On the other side, claudin 18.2 activation sustains tumor growth and distant dissemination, effects reversed by LILRB2 blockade. Consistently, in human pancreatic adenocarcinoma cohorts, combined low claudin 18.2 and LILRB2 tumor expression correlates with a more favorable overall survival, underscoring the clinical relevance of this axis as additional tumor-intrinsic immune evasion mechanism [14].

### Metabolic rewiring and adaptation to tumor-promoting conditions

LILRBs pathways support tumor progression even through metabolic rewiring. In triple-negative breast cancer, LILRB2 and PIRB enhance aerobic glycolytic metabolism by upregulating the glucose transporter GLUT3 and the glycolytic enzyme PKM2, thereby sustaining proliferative and invasive behavior. Importantly, blockade of GLUT3 and PKM2 abrogates these effects, demonstrating that LILRB-driven tumor progression is functionally dependent on glycolytic reprogramming. Mechanistically, LILRB2 activates the AKT-mTOR pathway, with a lesser contribution from ERK, to drive GLUT3- and PKM2-dependent glycolysis, whereas pathway inhibition abrogates these effects [85]. In parallel, LILRB2 signaling has been linked to lipid metabolic programs, including enhanced fatty acid synthesis and lipid accumulation, which directly contribute to tumor growth and T-cell senescence [90]. These observations are conceptually important because they place LILRB receptors at the intersection of immune regulation and metabolic adaptation. Rather than acting only through inhibitory phosphatase signaling, tumor-cell LILRBs appear capable of stabilizing broader anabolic programs that support survival under hostile microenvironmental conditions.

### Angiogenesis, invasion, and communication with the microenvironment

In addition to metabolic rewiring, LILRB receptors contribute to aberrant angiogenic programs. Specifically, LILRB2 signaling in NSCLC and colorectal cancer increases microvessel density and enhances endothelial migration and tube formation. Mechanistically, LILRB2 induces ERK-dependent upregulation of pro-angiogenic factors, including VEGF-A and MMP-9, a program further amplified by ANGPTL2 [95, 96]. Consistently, LILRB2-dependent ERK activation also drives VEGF-C expression, highlighting a broader role for this pathway in coordinating pro-angiogenic factors [97]. Beyond matrix metalloproteinases, ANGPTL2–LILRB2 signaling further promotes tumor dissemination through additional mechanisms. Tumor cells establish an autocrine loop by secreting ANGPTL2, which engages LILRB2 to drive epithelial-mesenchymal transition. In pancreatic cancer, silencing ANGPTL2 or blocking LILRB2 reduces mesenchymal features as well as proliferation and migration [88]. Indeed, ANGPTL2-activated LILRB2 promotes cell motility through interaction with integrin ITGB3 [98]. Collectively, these findings indicate that LILRB receptors function not only as intracellular amplifiers of malignancy but also as mediators of stromal remodeling and metastatic progression.

### LILRB family in hematological malignancies

Hematological malignancies are a heterogeneous group of lymphoproliferative disorders involving both lymphoid and myeloid cells, including chronic lymphocytic leukemia (CLL), acute myeloid leukemia (AML), B-cell lymphomas, and multiple myeloma (MM), in which LILRB family receptors are frequently dysregulated and exert both immunoregulatory and cell-intrinsic functions. Notably, LILRB3 and LILRB4 are selectively expressed in CLL and AML compared with normal cells, indicating their pivotal pro-tumoral role and their potential as therapeutic targets [30, 99]. LILRB4 directly contributes to tumor survival through BCR inhibition and activation of the oncogenic AKT/mTOR and STAT pathways [100]. Similarly, LILRB3 contributes to leukemic progression and immune resistance through the TRAF2-cFLIP-NF-kB axis, and its inhibition with antagonistic antibodies has been shown to arrest AML development [101]. Because of its pronounced expression in B-cell lymphomas, LILRB1 has recently been described as a novel marker of neoplastic B lymphoblasts [102] and as a driver of tumor progression through the CREB-SORBS3 pathway [103]. Likewise, in MM, LILRB1 promotes proliferation and survival of malignant cells through the GATA2-SAGE1 axis while also regulating lipid metabolism via the LDLR–LDLRAP1 complex, thereby protecting cells from ferroptosis [104]. Overall, the available evidence supports a model in which LILRBs contribute to hematological tumorigenesis through the dual action of immune suppression and activation of pro-tumorigenic signaling.

### A unifying model of tumor-intrinsic LILRB activity

Taken together, the available evidence supports a unifying model in which tumor-cell LILRB receptors act as coordinators of cancer cell fitness. Their tumor-intrinsic activity integrates proliferative signaling, metabolic plasticity, angiogenic and invasive programs, and active immune evasion. This distinguishes the LILRB axis from more conventional checkpoint pathways and reinforces the view that these receptors are not simply immune suppressive molecules ectopically expressed by tumors, but multifunctional regulators of malignant progression. This broader perspective has important therapeutic implications. If LILRB receptors simultaneously sustain tumor growth and protect tumors from immune elimination, then their targeting may provide a dual benefit: direct impairment of cancer cell fitness and indirect restoration of antitumor immunity.

## Therapeutic implications

### Monoclonal antibodies and combination strategies

**Fig. 2:**
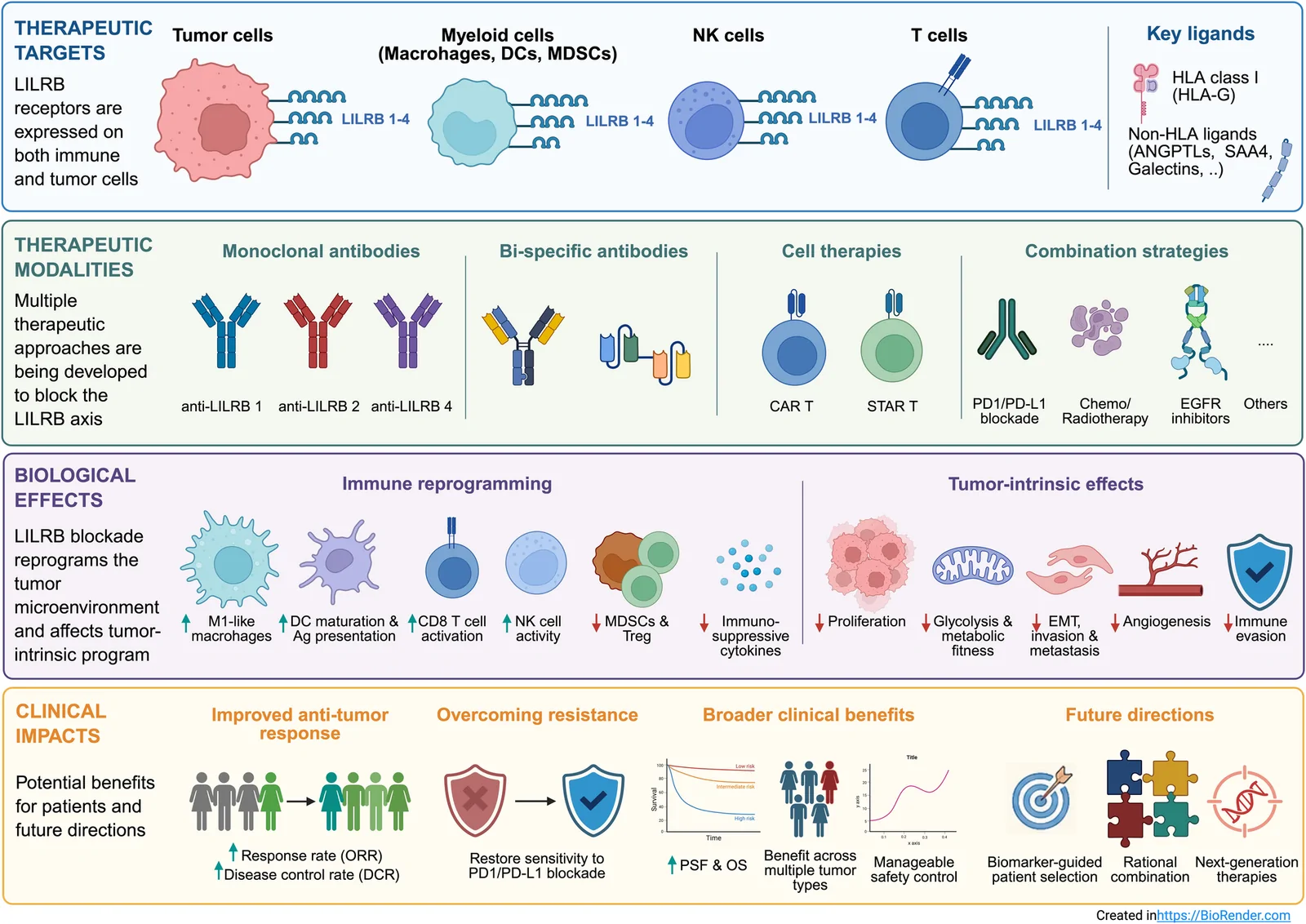
Therapeutic targeting of the LILRB axis reprograms tumor and immune compartments. LILRB-directed therapies target both immune and tumor compartments through monoclonal antibodies, bispecific antibodies, cell-based therapies, and rational combination approaches. By simultaneously reprogramming suppressive myeloid cells, restoring T- and NK-cell activity, and inhibiting tumor-intrinsic signaling, LILRB blockade promotes durable anti-tumor immunity while reducing tumor fitness. Ongoing clinical development suggests that targeting the LILRB axis may overcome resistance to conventional immune checkpoint blockade and expand the efficacy of cancer immunotherapy through biomarker-guided combination strategies. Figure created in https://BioRender.com

Targeting LILRB receptors represents a strategy to reprogram both immune and tumor compartments. Monoclonal antibodies against LILRB1, LILRB2, and LILRB4 restore myeloid-cell activation, enhance antigen presentation, and promote T- and NK-cell responses, as summarized in Table 1. LILRB blockade consistently restores macrophage phagocytosis of HLA-G^+^ and patient-derived tumor cells [105] and rewires suppressive M2-like macrophages and tolerogenic DCs toward pro-inflammatory, antigen-presenting phenotypes by upregulating proteins involved in antigen presentation, cell adhesion, and co-stimulation [44, 106, 107]. Collectively, these studies support the clinical feasibility of targeting LILRB receptors to reprogram myeloid-driven immunosuppression. Blockade of LILRB1 with monospecific antibodies BND-22 and B1M023 [105, 108] directly potentiates T- and NK-cell effector functions, including proliferation, IFNγ production, and cytotoxic activity (Fig. 2).

**Table 1:** Overview of monospecific therapeutic antibodies targeting LILRB family members currently under preclinical and clinical development in solid and hematological malignancies. The table summarizes target specificity, antibody format, and IgG subclass, available in vivo preclinical evidence, ongoing or completed clinical trials, types of tumors in which the treatments were investigated, patient enrollment, treatment regimens, and current clinical status. Most agents are primarily evaluated in advanced or metastatic solid tumors, either as monotherapy or in combination with immune checkpoint inhibitors, chemotherapy, or targeted agents. Several studies have been completed or terminated for strategic pipeline reasons unrelated to safety, whereas others remain active or recruiting

**Monoclonal antibodies targeting LILRBs**								
	Target	Name (IgG subclass)	Pre-clinical in vivo (tumors tested)	Clinical trial (ID)	Phase (types of tumors)	# patients	Treatments investigated	Status
monospecific antibodies	LILRB1	ADA-011	-	NCT05601219	phase I (advanced/ recurrent solid)	46	1) ADA-011 2) ADA-011 + anti PD-L1	terminated (non-safety related) (2024)
		AGEN-1571 (IgG4) [109]	-	NCT05377528	phase I (advanced/ recurrent solid)	22	1) AGEN1571 2) AGEN1571 + balstilimab (anti PD-1) + botensilimab (anti CTLA-4)	completed (2024)
		B1M023 [108]	-	-	-	-	-	-
		BND-22 (IgG4) [105]	xenograft cell lines (melanoma, colon, head and neck)	NCT04717375 [110]	phase I / II [advanced/metastatic solid (breast, cervical, CRC, adenocarcinoma or SCC of the esophagus, gastric, H&N SCC, HCC, gallbladder, NSCLC, RCC, SCC of the skin, PDA, ovarian and urothelial)]	125	1) BND-22 2) BND-22 + pembrolizumab (anti PD-1) 3) BND-22 + cetuximab (anti EGFR) 4) BND-22 + pembrolizumab/ carboplatin/pemetrexed (NSCLC, gastric, esophagus, CRC)	terminated due to pipeline changes (non-safety related) (2025)
	LILRB2	AZD2796 (IgG1) [107]	xenograft cell lines (melanoma, bronchioalveolar carcinoma, lung adenocarcinoma)	1009199	phase I/IIa (advanced solid)	46	AZD2796	ongoing
		CHS-1000 (IgG1) [111]	-	NCT06389526	phase I (advanced/metastatic extracranial solid)	48	1) CHS-1000 2) CHS-1000 + toripalimab (anti PD-1)	active, not yet recruiting
		ES009 (IgG4) [112]	-	NCT06007482 [113]	phase I (advanced/metastatic solid, refractory or relapsed)	12	ES009	completed (2025)
		IO-108 (IgG4) [114]	LILRB2-transgenic murine cell line (Lewis lung carcinoma)	NCT05054348 [115]	phase Ib (advanced/metastatic solid)	91	1) IO-108 2) IO-108 + pembrolizumab (anti PD-1) 3) IO-108 + cemiplimab (anti PD-1)	completed (2024)
				NCT05508100	phase I (advanced/metastatic solid)	38	1) IO-108 2) IO-108 + pembrolizumab (anti PD-1) 3) IO-108 + tislelizumab (anti PD-1)	completed (2024)
monospecific antibodies				NCT04524871	phase Ib/II (advanced or metastatic, and/or unresectable HCC)	518	Umbrella study, the treatment involving IO-108:- IO-108 (1800 mg)- IO-108 (1200 mg)+ atezolizumab (anti PD-L1)+ bevacizumab (anti VEGF-A)	active, recruiting
		JTX-8064 (IgG1) [44]	ex vivo human tumors	NCT04669899 [116]	phase I/II [advanced/metastatic extracranial solid (ovarian cancer, RCC, TNBC, SCC H&N, SCC of the skin, pleomorphic sarcoma, liposarcoma, BTC)]	190	1) JTX-8064 2) JTX-8064 + pimivalimab (anti PD-1)	completed (2023)
		MK-4830 (IgG4) [117]	xenograft cell line (melanoma)	NCT03564691 [118]	phase I [advanced/metastatic solid (PDA, glioblastoma multiforme, H&N SCC, NSCLC, RCC, gastric, gastroesophageal, ovarian, fallopian tube, primary peritoneal, TNBC, mesothelioma)]	470	1) MK-4830 2) MK-4830 + pembrolizumab (anti PD-1) 3) MK-4830 + pembrolizumab + carboplatin + pemetrexed (NSCLC) 4) MK-4830 + pembrolizumab + lenvatinib (VEGFRi)(RCC) 5) MK-4830 + pembrolizumab + paclitaxel (ovarian, TNBC) 6) MK-4830 + pembrolizumab + cisplatin + pemetrexed (mesothelioma) 7) co-formulation MK-4830 + pembrolizumab	completed (2025)
				NCT06413095	early phase I [primary/recurrent/metastatic solid (H&N SCC, STS)]	13	tumor or regional node dissection + 2/4 days prior intratumoral injection of pembrolizumab (anti PD-1) + MK-4830 or pembrolizumab + MK-0482	completed (2023)
				NCT05446870	phase II (stage III / stage IV HGSOC primary peritoneal fallopian tube)	160	Pembrolizumab (anti PD-1) + paclitaxel + carboplatin + avastin + docetaxel + MK-4830	completed (2024)
		OR-502 (IgG1) [119]	xenograft cell line (melanoma)	NCT06090266 [120, 121]	phase I/II [carcinoma, sarcoma, melanoma with progressive locally advanced/metastatic disease with prior PD-L1 (B2-B5 arms) or platinum-based therapy (B3 arm)]	168	1) OR-502 2) OR-502 + cemiplimab (anti PD-1)	active, recruiting
monospecific antibodies	LILRB4	h128-3(IgG1) [106]	LILRB4-transgenicmurine cell line (Acute myeloid leukemia)	-	-	-	-	-
		IO-202 (IgG1) [122]	LILRB4-transgenic murine cell line (Lewis lung carcinoma)	NCT04372433 [123, 124]	phase I (AML with myelomonocitic or monoblastic/monocytic differentiation with refractory or relapsed disease CMML)	67	1) IO-202 2) IO-202 + azacitidine 3) IO-202 + azacitidine + venetoclax	completed (2025)
				NCT05309187	phase I (advanced/metastatic solid)	22	1) IO-202 2) IO-202 + pembrolizumab (anti PD-1)	completed (2024)
		MK-0482 (IgG4)	-	NCT03918278 [125, 126]	phase I [advanced/metastatic solid (TNBC, glioblastoma, metastatic PDA, STS, NSCLC)]	222	1) MK-0482 2) MK-0482 + pembrolizumab (anti PD-1) 3) MK-0482 + pembrolizumab + paclitaxel (TNBC) 4) MK-0482 + pembrolizumab + Nab-paclitaxel + gemcitabine (PDA) 5) MK-0482 + pembrolizumab + carboplatin + pemetrexed (NSCLC)	completed (2025)
				NCT05038800	phase I (AML with myelomonocitic or monoblastic/monocytic differentiation with refractory or relapsed disease)	13	MK-0482 in 5 different doses (7.5 / 25 / 75 / 225 / 750 mg)	terminated for business reason (2025)
		NGM-831 (IgG1) [127]	-	NCT05215574	phase I (advanced/metastatic solid)	130	1) NGM-831 2) NGM-831 + pembrolizumab (anti PD-1) 3) NGM-831 + NGM438 (LAIR1i) + pembrolizumab (anti PD-1)	active, not recruiting

Importantly, these effects are amplified when combined with PD-1/PD-L1 blockade; indeed, LILRB1 upregulation has been observed in a subset of patients receiving ICB [105], suggesting that LILRB signaling contributes to resistance to conventional checkpoint inhibitors [44, 107, 127]. LILRB1 blockade also potentiates antibody-dependent cellular phagocytosis of EGFR^+^ cancer cells when combined with cetuximab [105] and can enhance radiotherapy efficacy, as shown in a radiation-resistant Lewis lung carcinoma (LLC) model [122]. These immunostimulatory effects translate into reduced tumor growth and metastatic spread in humanized mouse models of multiple cancer types (Table 1). Beyond monospecific antibodies, bispecific and multivalent approaches targeting multiple LILRB receptors have also been developed (Table 2). NGM-707 and IOMX-0675 are dual LILRB1/LILRB2 antagonistic antibodies that showed in vitro and in vivo efficacy and synergy with PD-1 blockade [128, 129]. A second class of bispecific agents simultaneously targets the LILRB and PD-1/PD-L1 axes: CDX-585 is a bispecific LILRB2/PD-1 antibody that drives inflammatory reprogramming and achieves superior tumor control compared with single-agent approaches [130]. Similarly, IOS-1002 engages LILRB1, LILRB2, and the NK-cell inhibitory receptor KIR3DL1 [131], whereas an anti-LILRB4 antibody has been coupled to anti-CD3 to efficiently kill AML cells as demonstrated both in vitro and in NSG mouse models [132].

**Table 2:** Bispecific antibodies targeting LILRB family receptors. The table reports bispecific molecules simultaneously targeting LILRB receptors together with additional inhibitory or activating immune receptors, including PD-1, PD-L1, KIR3DL1, and CD3. Information regarding preclinical validation, clinical trial identifiers, types of tumors in which the treatments were investigated, treatment combinations, enrollment, and development status is summarized

**Bispecific antibodies targeting LILRBs**								
	Target	Name (IgG subclass)	Pre-clinical in vivo (tumors tested)	Clinical trial (ID)	Phase (types of tumors)	# patients	Treatments investigated	Status
Bispecific antibodies	LILRB1/2	IOMX-0675 (IgG1) [129]	xenograft cell lines (melanoma)	2024-517449-14-00	phase I/II [advanced/metastatic solid (NSCLC, gastric, gastroesophageal, mesothelioma, RCC, CRC) previously treated]	-	1) IOMX-0675 2) IOMX-0675 + pembrolizumab (anti PD-1)	active
		NGM-707 [128]	-	NCT04913337	phase I/II (advanced/metastatic solid)	179	1) NGM-707 2) NGM-707 + pembrolizumab (anti PD-1)	active, not recruiting
		PF-07826390 (IgG1)	-	NCT06546553	phase I [advanced/metastastic (NSCLC, CRC, RCC)]		PF-07826390 + sasalinmab (anti PD-1)	terminated due to pipeline changes (non-safety related) (2025)
	LILRB2/PD1	CDX-585 (IgG1) [130]	xenograft cell lines (melanoma)	NCT05788484	phase I (advanced/metastatic extracranial solid)	20	CDX-585	completed (2025)
	LILRB2/PDL1	SPX-303 (IgG4) [133]	yes (not specified)	NCT06259552	phase I (advanced/metastatic solid)	232	SPX-303	active, recruiting
	LILRB1/2/KIR3DL1	IOS-1002 (IgG4) [131]	ex vivo human tumors (NSCLC)	NCT05763004	phase Ia/b (advanced/metastatic solid refractory or relapsed)	140	1) IOS-1002 2) IOS-10002 + pembrolizumab (anti PD-1)	active, recruiting
	LILRB4/CD3	IO-312 (IgG-like) [132]	xenograft cell lines (Acute myeloid leukemia)	-	-	-	-	-

### CAR-T targeting LILRB molecules

Chimeric Antigen Receptor (CAR)-T strategies targeting LILRB-expressing cells have primarily been explored in hematological malignancies, both in vitro and in xenograft preclinical models, underscoring their potential to target both tumor cells and immunosuppressive myeloid populations (Table 3). A second-generation CAR-T directed against LILRB3 not only displayed cytotoxicity against AML both in vitro and in vivo, but also increased expression of activation markers, such as CD25 and CD107 [134]. Similarly, second-generation LILRB4 CAR-T cells achieved near-complete eradication of monocytic AML in xenograft models without detectable toxicity [135]. Targeting flexibility and resistance may be further overcome through the STAR-T platform, a CD3-accessorized TCR in which the conventional variable regions are replaced with antigen-specific nanobodies against LILRB4; this platform has shown efficacy against in vivo MM models [136]. Finally, a second- generation CAR-T cell directed against LILRB1, evaluated in B-cell acute lymphoblastic leukemia (B-ALL) and B-cell non-Hodgkin lymphoma (B-NHL), malignancies frequently characterized by CD19 loss, showed efficacy comparable to that of CD19-directed CAR-T cells while retaining activity against CD19 CAR-T-resistant Raji tumor cells [137]. New frontiers in this space have been recently explored, including a bispecific LILRB3/LILRB4 CAR-T which was developed to overcome the heterogeneous and only partially overlapping expression of these two receptors across AML patients. In preclinical models, the dual-targeting construct achieved potent and dose-dependent cytotoxicity against AML both in vitro and in xenograft models, where a strong decrease of both subcutaneous and systemic tumor growth was observed alongside a significantly prolonged survival [138].

### Clinical trials

Monotherapy with LILRB-targeting antibodies in solid tumors has shown limited objective response rates (Tables 1, 2 and 3). Nevertheless, clinically meaningful disease control and durable responses have been observed, with rates often exceeding 50–70%, an important result in heavily pretreated patients previously exposed and resistant to ICB [113, 116]. Across early-phase studies, complete (CR) and partial (PR) responses have also been reported in melanoma, NSCLC, ovarian cancer, and Merkel cell carcinoma [115, 118, 120]. By contrast, combination strategies involving PD-1/PD-L1 inhibitors have shown enhanced efficacy, with higher objective response rates and improved disease control in patients previously resistant to immunotherapy. The anti-LILRB2 antibody MK-4830 combined with pembrolizumab (anti-PD-1) achieved an overall response rate (ORR) of 24%, including one CR and seven PRs, with durable disease control in patients with advanced solid tumors, half of whom were refractory to ICB [118]. In metastatic pancreatic cancer, the anti-LILRB4 antibody MK-0482 added to pembrolizumab plus gemcitabine and nab-paclitaxel resulted in an ORR of 43% and a disease control rate of 84%, exceeding historical chemotherapy results [125]. Importantly, these findings suggest that LILRB-targeting strategies may complement PD-1/PD-L1 blockades by acting on non-redundant immune compartments. In hematological malignancies, LILRB4 targeting has shown robust clinical activity: IO-202 plus azacitidine achieved high response rates in AML, including durable remissions and frequent bridging to transplantation [123]. Early cellular therapies further validate this axis, as LILRB4-directed STAR-T cells induced objective responses in heavily pretreated relapsed AML. Notably, responses correlated with near-complete elimination of LILRB4^+^ blasts [139].

**Table 3:** Cell-based immunotherapeutic strategies targeting LILRB family members. The table summarizes preclinical and clinical development of chimeric antigen receptor T-cell (CAR-T) and synthetic T-cell receptor and antigen receptor (STAR-T) approaches directed against LILRB family proteins, mainly in acute myeloid leukemia (AML), chronic myelomonocytic leukemia (CMML), and multiple myeloma. Reported information includes preclinical tumor models, clinical trial identifiers, treatment regimens, patient numbers, and current study status

**Cell-based therapy targeting LILRBs**							
	**Target**	**Pre-clinical ** ***in vivo*** **(tumors tested)**	**Clinical trial ** **(ID)**	**Phase** **(types of tumors)**	**# patients**	**Treatments investigated**	**Status**
CAR-T	LILRB1 CART [137]	xenograft cell lines (Acute myeloid leukemia)	-	-	-	-	-
	LILRB3 CART [134]	xenograft cell lines (Acute myeloid leukemia)	-	-	-	-	-
	LILRB4 CART [135]	xenograft cell lines	NCT04803929	early phase I (AML M4-M5 subtypes refractory or relapsed)	25	anti LILRB4 CART	active, recruiting
	LILRB4 START [136]	xenograft cell lines (Multiple myeloma)	NCT05518357	phase I (AML refractory or relapsed)	2	cytoreductive chemotherapy+ anti LILRB4 START	completed(2022)
			NCT05548088 [134]	phase I (AML and CMML refractory or relapsed)	11	cytoreductive chemotherapy+ anti LILRB4 START	completed(2024)
			NCT05739409	(AML and CMML refractory or relapsed)	10	cytoreductive chemotherapy+ anti LILRB4 START at five escalating doses	unknown status
	LILRB3/4 CART [138]	Xenograft cell lines (Acute myeloid leukemia)	-	-	-	-	-

Collectively, LILRB blockade may help overcome resistance to immune checkpoint inhibitors. A key conceptual advance is that LILRB targeting differs from classical checkpoint blockade. Rather than acting solely on T cells, LILRB inhibition reprograms the tumor ecosystem by simultaneously affecting myeloid cells, tumor cells, and stromal interactions (Fig. 2).

### Challenges and limitations of current LILRB-targeted therapies

Despite encouraging preclinical results, several clinical challenges remain unresolved. Shared ligands across LILRB family members complicates the design of receptor-selective antagonists. Reassuringly, however, specificity data from several anti-LILRB candidates currently under investigation are encouraging: the anti-LILRB4 antibody 128-3 showed no cross-reactivity against the other ten LILR family members despite their close phylogenetic relationship, especially within the D1 domain [106]. Similarly, the anti-LILRB2 antibody AZD2796 did not bind to closely related receptors sharing high sequence homology in their extracellular Ig domains (LILRB1, LILRA1-3), nor to the remaining LILR family members, and IOS-1002 showed no off-target binding and no significant cytokine release in primary PBMC assays, limiting cytokine storm-like toxicity [107, 131]. Consistent with this, treatment-related adverse events (TRAEs) reported across early-phase trials targeting different LILRB family members have generally been low-grade, with low rates of grade *≥* 3 events, suggesting that the treatment windows used so far do not drive an autoimmune-like state of immune hyperactivation [110, 124]. Although fatal events related to disease progression were reported, as expected in heavily pretreated populations with advanced malignancies, deaths attributed to TRAEs were strongly excluded [116, 118].

Resistance mechanisms have also emerged: LILRB1 has been reported to be upregulated in a substantial fraction of melanoma patients following anti-PD-1 therapy, suggesting a compensatory resistance mechanism to single-checkpoint blockade that can be overcome following LILRB-targeted approaches [105]. Similarly, LILRB4-targeting antibody can circumvent resistance of LILRB4^+^ AML to venetoclax alone or combined with azacitidine [124].

Importantly, the current limitations lie in the clinical studies conducted so far, which are all early-stage studies limited to small patient cohorts, often heavily pretreated and refractory to prior lines of therapy. The response rates observed in these populations are nonetheless encouraging and suggest that anti-LILRB antibodies may indeed be capable of overcoming such treatment resistance. Larger dedicated clinical studies and pre-clinical models will be needed to address several open questions: (I) at what dose and after what duration of treatment immune-mediated off-target events may emerge; (II) whether tumors can deploy compensatory mechanisms, such as upregulation of alternative LILRB isoforms with overlapping functional roles to bypass single-receptor blockade; (III) whether combination strategies with approved therapies could lead to reduce dose administration and mitigation of off-target adverse effects risk; (IV) whether non-antibody based targeting strategies could offer viable alternative or complements to current approaches. In this regard, a biomimetic nanoplatform engineered to penetrate blood-brain-barrier and specifically deliver siLILRB3 to M2 macrophages has recently been developed for glioma, achieving selective LILRB3 silencing inside the TME and a shift toward a “hotter” immune landscape enriched in M1 and effector T and NKT cells, ultimately reducing tumor growth in preclinical models [81]. This suggests how new strategies, such as the RNA interference-based or small molecule inhibitor approaches, may eventually broaden the toolkit beyond monoclonal antibodies.

## Open questions and future directions

Several key questions remain unresolved. First, it remains unclear whether ligands capable of engaging multiple LILRB isoforms, such as HLA class I molecules or ANGPTLs, drive conserved or receptor-specific functional programs, and how these effects are shaped by the cellular context in which LILRB receptors are expressed. Second, the relative contribution of extrinsic versus tumor-intrinsic signaling to disease progression remains to be defined. Nonetheless, there is little doubt that these receptors, when directly expressed by tumor cells, affect tumor growth, as demonstrated by several groups and supported by our recent unpublished data. In a simple crossed orthotopic experiment, PIRB-knockout pancreatic adenocarcinoma (PDA) cells injected into wild-type mice grew less than their wild-type PDA counterparts. As expected, wild-type PDA cells also grew less when injected into PIRB-knockout mice (*manuscript in preparation*). Third, LILRB polymorphisms, particularly LILRB2 and LILRB3, may influence receptor function and therapeutic response. Patients harboring LILRB-high tumors consistently exhibit reduced overall and progression-free survival. Moreover, the rs383369 (R20H) LILRB2 polymorphism has been associated with increased lymph-node infiltration in colorectal cancer, with the allele conferring additive risk and contributing to tumor aggressiveness [140]. Finally, it remains to be established whether targeting LILRB receptors can consistently overcome resistance to PD-1-based therapies across tumor types owing to their dual role in modulating the antitumor response extrinsically and cancer-cell behavior intrinsically.

## Conclusion

LILRB receptors define a class of innate immune checkpoints that extend beyond the classical T cell-centric paradigm of immune regulation. By integrating signals across immune, stromal, and tumor compartments, the LILRB/PIRB axis coordinates a multilayered program of immune suppression while simultaneously sustaining tumor cell fitness (Fig. 1). This dual extrinsic and intrinsic activity distinguishes LILRBs from established checkpoint pathways and provides a conceptual framework for understanding resistance to current immunotherapies, particularly in tumors characterized by strong myeloid-driven immunosuppression. From a translational perspective, targeting the LILRB axis offers a unique opportunity to simultaneously reprogram suppressive myeloid populations, restore effective T and NK cell responses, and directly impair tumor-intrinsic survival and metabolic programs (Fig. 2). Early clinical evidence, particularly in combination with PD-1/PD-L1 blockade, supports the feasibility of this approach and highlights its potential to overcome resistance in patients who do not benefit from current therapies. Future efforts will need to define the relative contribution of immune versus tumor-intrinsic LILRB signaling across tumor types, the impact of ligand diversity and receptor polymorphisms, and optimal strategies for therapeutic targeting and combination approaches. Addressing these questions will be critical to fully exploit the LILRB axis as a next-generation immunotherapeutic target. Collectively, current evidence positions LILRB receptors as central regulators of the tumor-immune interface and supports their development as dual-action targets capable of overcoming resistance and reshaping cancer immunotherapy.

## Data Availability

No datasets were generated or analysed during the current study.

## Abbreviations

AML: Acute myeloid leukemia
AKT: AKT kinase is also known as protein kinase B
ANGPTLs: Angiopoietin like protein
APC: Antigen presenting cells
APOE: Apolipoprotein E
B7-H3: B7 Homolog 3
B-ALL: B-cell acute lymphoblastic leukemia
BCR: B cell receptor
B-NHL: B-cell non-Hodgkin lymphoma
BTC: Biliary tract cancer
CAMK: Calcium/calmodulin-dependent protein kinase
CAR-T: Chimeric Antigen Receptor - T
cFLIP: Cellular FLICE-inhibitory protein
CLL: Chronic lymphocytic leukemia
CMML: Chronic myeloid monocytic leukemia
CR: Complete response
CRC: Colon rectal cancer
CREB: CAMP response element-binding protein
CTLA-4: Cytotoxic T lymphocyte–associated protein 4
DAMPs: Damage-Associated Molecular Patterns
DC: Dendritic cells
EGFR: Epithelial growth factor receptor
ERK: Extracellular signal-Regulated Kinase
FcαR: Fragment crystallizable IgA receptor
FcεR: Fragment crystallizable IgE receptor
FcγR: Fragment crystallizable IgG receptor
FOXP3: Forkhead Box Protein 3
GATA2: GATA-binding factor 2
GLUT3: Glucose transporter 3
GzmB: Granzyme B
HCC: Hepatocellular carcinoma
HGSOC: High grade serous ovarian cancer
HLA: Human Leukocyte antigen
H&N: Head and neck carcinoma
ICB: Immune checkpoint blockade
IL: Interleukin
IFNγ: Interferon gamma
ITAMs: Immunoreceptor tyrosine-based activation motifs
ITIMs: Immunoreceptor tyrosine-based inhibitory motifs
ITSM: Immunoreceptor tyrosine-based switch motif
ITGB3: Integrin beta 3
KIR3DL1: Killer cell Immunoglobulin-like Receptor, Three Ig domains and Long cytoplasmic tail1
LDLR: Low-Density Lipoprotein Receptor
LDLRAP1: Low-Density Lipoprotein Receptor Adaptor Protein 1
LILR: Leukocyte immunoglobulin-like receptor
LLC: Lewis lung carcinoma
M2-like: Macrophage type 2-like
MDSCs: Myeloid-derived suppressor cells
MHC: Major histocompatibility complex
MM: Multiple myeloma
MMP: Matrix metalloproteinases
mTORC1: Target of rapamycin complex 1
NF-κB: Nuclear factor kappa-light-chain-enhancer of activated B cells
NK: Natural killer cells
NKT: Natural Killer T cells
NSCLC: Non-small cell lung cancer
NSG: NOD scid gamma mouse
ORR: Overall response rate
PAMPs: Pathogen-Associated Molecular Patterns
PD-1: Programmed cell death protein 1
PDA: Pancreatic ductal adenocarcinoma
PD-L1: Programmed death ligand 1
PI3K: Phosphatidylinositol 3-kinases
PIRB: Paired Immunoglobulin-like receptor B
PR: Partial response
PKM2: Pyruvate kinase muscle 2
RCC: Renal cell carcinoma
SAA4: Serum amyloid A4
SAGE1: Sarcoma antigen 1
SCC: Squamous cell carcinoma
SEMA-4A: Semaphorin-4 A
SHIP: Src homology 2 domain-containing inositol 5-phosphatase
SHP-1/2: Src homology region 2 domain-containing phosphatase-1/2
SORBS3: Sorbin And SH3 Domain Containing 3
STAR-T: Synthetic T-Cell Receptor and Antigen Receptor-T
STAT: Signal Transducer and Activator of Transcription
STS: Soft-tissue sarcoma
TCR: T cell receptor
TGFβ: Transforming growth factor beta
Th: T helper cell
TLR: Toll-like receptor
TME: Tumor microenvironment
TNBC: Triple negative breast cancer
TNF: Tumor necrosis factor
Tr1: Type 1 regulatory cells
TRAF: TNF Receptor-Associated Factors
Treg: Regulatory T cells
VEGF-A: Vascular endothelial growth factor-A
